# Ten Simple Rules for Writing a Literature Review

**DOI:** 10.1371/journal.pcbi.1003149

**Published:** 2013-07-18

**Authors:** Marco Pautasso

**Affiliations:** 1 Centre for Functional and Evolutionary Ecology (CEFE), CNRS, Montpellier, France; 2 Centre for Biodiversity Synthesis and Analysis (CESAB), FRB, Aix-en-Provence, France; University of California San Diego, United States of America

Literature reviews are in great demand in most scientific fields. Their need stems from the ever-increasing output of scientific publications [1]. For example, compared to 1991, in 2008 three, eight, and forty times more papers were indexed in Web of Science on malaria, obesity, and biodiversity, respectively [2]. Given such mountains of papers, scientists cannot be expected to examine in detail every single new paper relevant to their interests [3]. Thus, it is both advantageous and necessary to rely on regular summaries of the recent literature. Although recognition for scientists mainly comes from primary research, timely literature reviews can lead to new synthetic insights and are often widely read [4]. For such summaries to be useful, however, they need to be compiled in a professional way [5].

When starting from scratch, reviewing the literature can require a titanic amount of work. That is why researchers who have spent their career working on a certain research issue are in a perfect position to review that literature. Some graduate schools are now offering courses in reviewing the literature, given that most research students start their project by producing an overview of what has already been done on their research issue [6]. However, it is likely that most scientists have not thought in detail about how to approach and carry out a literature review.

Reviewing the literature requires the ability to juggle multiple tasks, from finding and evaluating relevant material to synthesising information from various sources, from critical thinking to paraphrasing, evaluating, and citation skills [7]. In this contribution, I share ten simple rules I learned working on about 25 literature reviews as a PhD and postdoctoral student. Ideas and insights also come from discussions with coauthors and colleagues, as well as feedback from reviewers and editors.

## Rule 1: Define a Topic and Audience

How to choose which topic to review? There are so many issues in contemporary science that you could spend a lifetime of attending conferences and reading the literature just pondering what to review. On the one hand, if you take several years to choose, several other people may have had the same idea in the meantime. On the other hand, only a well-considered topic is likely to lead to a brilliant literature review [8]. The topic must at least be:

- interesting to you (ideally, you should have come across a series of recent papers related to your line of work that call for a critical summary),
- an important aspect of the field (so that many readers will be interested in the review and there will be enough material to write it), and
- a well-defined issue (otherwise you could potentially include thousands of publications, which would make the review unhelpful).

Ideas for potential reviews may come from papers providing lists of key research questions to be answered [9], but also from serendipitous moments during desultory reading and discussions. In addition to choosing your topic, you should also select a target audience. In many cases, the topic (e.g., web services in computational biology) will automatically define an audience (e.g., computational biologists), but that same topic may also be of interest to neighbouring fields (e.g., computer science, biology, etc.).

## Rule 2: Search and Re-search the Literature

After having chosen your topic and audience, start by checking the literature and downloading relevant papers. Five pieces of advice here:

- keep track of the search items you use (so that your search can be replicated [10]),
- keep a list of papers whose pdfs you cannot access immediately (so as to retrieve them later with alternative strategies),
- use a paper management system (e.g., Mendeley, Papers, Qiqqa, Sente),
- define early in the process some criteria for exclusion of irrelevant papers (these criteria can then be described in the review to help define its scope), and
- do not just look for research papers in the area you wish to review, but also seek previous reviews.

**Figure 1:**
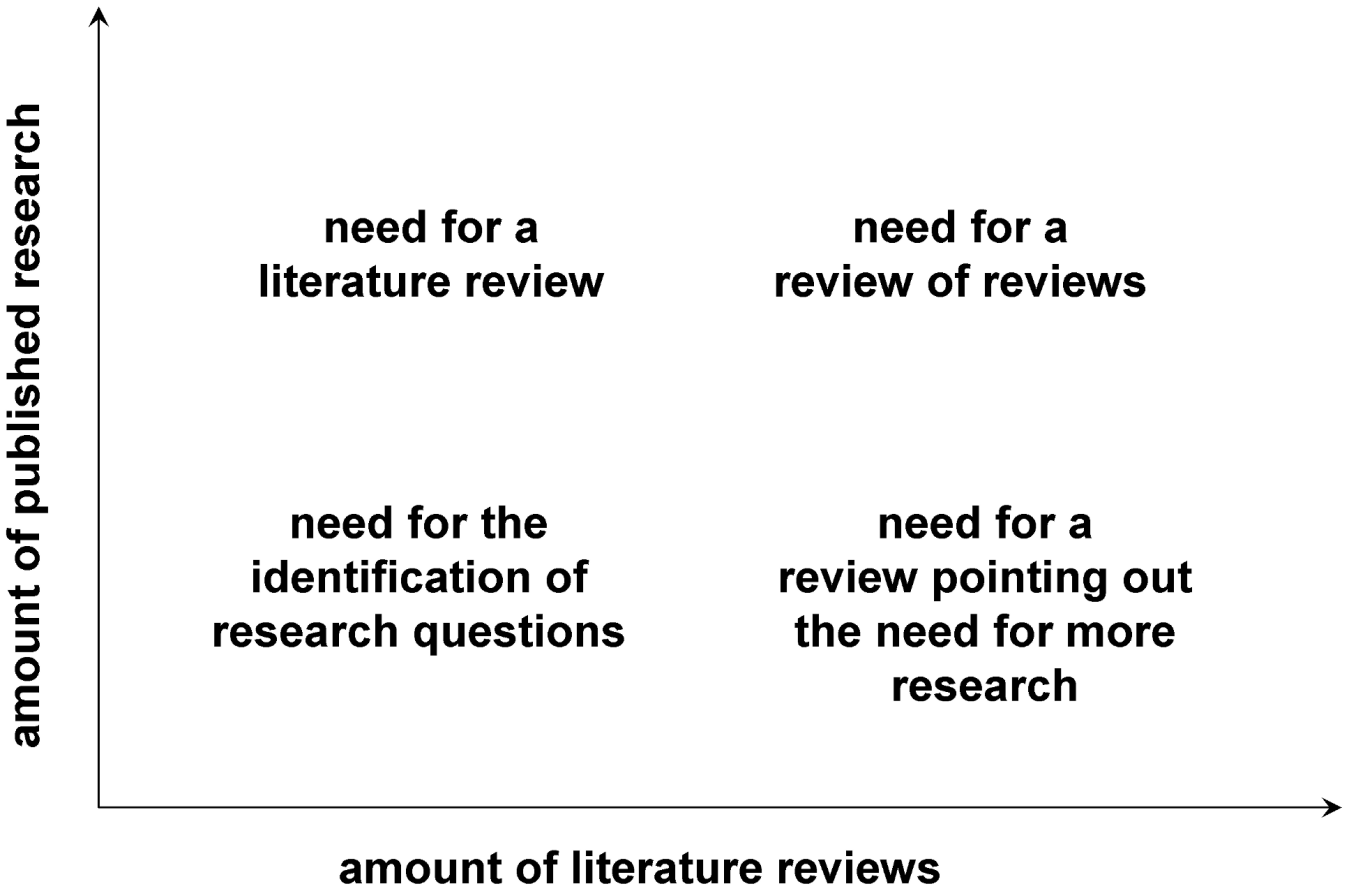
A conceptual diagram of the need for different types of literature reviews depending on the amount of published research papers and literature reviews. The bottom-right situation (many literature reviews but few research papers) is not just a theoretical situation; it applies, for example, to the study of the impacts of climate change on plant diseases, where there appear to be more literature reviews than research studies [33].

The chances are high that someone will already have published a literature review (Figure 1), if not exactly on the issue you are planning to tackle, at least on a related topic. If there are already a few or several reviews of the literature on your issue, my advice is not to give up, but to carry on with your own literature review,

- discussing in your review the approaches, limitations, and conclusions of past reviews,
- trying to find a new angle that has not been covered adequately in the previous reviews, and
- incorporating new material that has inevitably accumulated since their appearance.

When searching the literature for pertinent papers and reviews, the usual rules apply:

- be thorough,
- use different keywords and database sources (e.g., DBLP, Google Scholar, ISI Proceedings, JSTOR Search, Medline, Scopus, Web of Science), and
- look at who has cited past relevant papers and book chapters.

## Rule 3: Take Notes While Reading

If you read the papers first, and only afterwards start writing the review, you will need a very good memory to remember who wrote what, and what your impressions and associations were while reading each single paper. My advice is, while reading, to start writing down interesting pieces of information, insights about how to organize the review, and thoughts on what to write. This way, by the time you have read the literature you selected, you will already have a rough draft of the review.

Of course, this draft will still need much rewriting, restructuring, and rethinking to obtain a text with a coherent argument [11], but you will have avoided the danger posed by staring at a blank document. Be careful when taking notes to use quotation marks if you are provisionally copying verbatim from the literature. It is advisable then to reformulate such quotes with your own words in the final draft. It is important to be careful in noting the references already at this stage, so as to avoid misattributions. Using referencing software from the very beginning of your endeavour will save you time.

## Rule 4: Choose the Type of Review You Wish to Write

After having taken notes while reading the literature, you will have a rough idea of the amount of material available for the review. This is probably a good time to decide whether to go for a mini- or a full review. Some journals are now favouring the publication of rather short reviews focusing on the last few years, with a limit on the number of words and citations. A mini-review is not necessarily a minor review: it may well attract more attention from busy readers, although it will inevitably simplify some issues and leave out some relevant material due to space limitations. A full review will have the advantage of more freedom to cover in detail the complexities of a particular scientific development, but may then be left in the pile of the very important papers “to be read” by readers with little time to spare for major monographs.

There is probably a continuum between mini- and full reviews. The same point applies to the dichotomy of descriptive vs. integrative reviews. While descriptive reviews focus on the methodology, findings, and interpretation of each reviewed study, integrative reviews attempt to find common ideas and concepts from the reviewed material [12]. A similar distinction exists between narrative and systematic reviews: while narrative reviews are qualitative, systematic reviews attempt to test a hypothesis based on the published evidence, which is gathered using a predefined protocol to reduce bias [13], [14]. When systematic reviews analyse quantitative results in a quantitative way, they become meta-analyses. The choice between different review types will have to be made on a case-by-case basis, depending not just on the nature of the material found and the preferences of the target journal(s), but also on the time available to write the review and the number of coauthors [15].

## Rule 5: Keep the Review Focused, but Make It of Broad Interest

Whether your plan is to write a mini- or a full review, it is good advice to keep it focused 16,17. Including material just for the sake of it can easily lead to reviews that are trying to do too many things at once. The need to keep a review focused can be problematic for interdisciplinary reviews, where the aim is to bridge the gap between fields [18]. If you are writing a review on, for example, how epidemiological approaches are used in modelling the spread of ideas, you may be inclined to include material from both parent fields, epidemiology and the study of cultural diffusion. This may be necessary to some extent, but in this case a focused review would only deal in detail with those studies at the interface between epidemiology and the spread of ideas.

While focus is an important feature of a successful review, this requirement has to be balanced with the need to make the review relevant to a broad audience. This square may be circled by discussing the wider implications of the reviewed topic for other disciplines.

## Rule 6: Be Critical and Consistent

Reviewing the literature is not stamp collecting. A good review does not just summarize the literature, but discusses it critically, identifies methodological problems, and points out research gaps [19]. After having read a review of the literature, a reader should have a rough idea of:

- the major achievements in the reviewed field,
- the main areas of debate, and
- the outstanding research questions.

It is challenging to achieve a successful review on all these fronts. A solution can be to involve a set of complementary coauthors: some people are excellent at mapping what has been achieved, some others are very good at identifying dark clouds on the horizon, and some have instead a knack at predicting where solutions are going to come from. If your journal club has exactly this sort of team, then you should definitely write a review of the literature! In addition to critical thinking, a literature review needs consistency, for example in the choice of passive vs. active voice and present vs. past tense.

## Rule 7: Find a Logical Structure

Like a well-baked cake, a good review has a number of telling features: it is worth the reader's time, timely, systematic, well written, focused, and critical. It also needs a good structure. With reviews, the usual subdivision of research papers into introduction, methods, results, and discussion does not work or is rarely used. However, a general introduction of the context and, toward the end, a recapitulation of the main points covered and take-home messages make sense also in the case of reviews. For systematic reviews, there is a trend towards including information about how the literature was searched (database, keywords, time limits) [20].

How can you organize the flow of the main body of the review so that the reader will be drawn into and guided through it? It is generally helpful to draw a conceptual scheme of the review, e.g., with mind-mapping techniques. Such diagrams can help recognize a logical way to order and link the various sections of a review [21]. This is the case not just at the writing stage, but also for readers if the diagram is included in the review as a figure. A careful selection of diagrams and figures relevant to the reviewed topic can be very helpful to structure the text too [22].

## Rule 8: Make Use of Feedback

Reviews of the literature are normally peer-reviewed in the same way as research papers, and rightly so [23]. As a rule, incorporating feedback from reviewers greatly helps improve a review draft. Having read the review with a fresh mind, reviewers may spot inaccuracies, inconsistencies, and ambiguities that had not been noticed by the writers due to rereading the typescript too many times. It is however advisable to reread the draft one more time before submission, as a last-minute correction of typos, leaps, and muddled sentences may enable the reviewers to focus on providing advice on the content rather than the form.

Feedback is vital to writing a good review, and should be sought from a variety of colleagues, so as to obtain a diversity of views on the draft. This may lead in some cases to conflicting views on the merits of the paper, and on how to improve it, but such a situation is better than the absence of feedback. A diversity of feedback perspectives on a literature review can help identify where the consensus view stands in the landscape of the current scientific understanding of an issue [24].

## Rule 9: Include Your Own Relevant Research, but Be Objective

In many cases, reviewers of the literature will have published studies relevant to the review they are writing. This could create a conflict of interest: how can reviewers report objectively on their own work [25]? Some scientists may be overly enthusiastic about what they have published, and thus risk giving too much importance to their own findings in the review. However, bias could also occur in the other direction: some scientists may be unduly dismissive of their own achievements, so that they will tend to downplay their contribution (if any) to a field when reviewing it.

In general, a review of the literature should neither be a public relations brochure nor an exercise in competitive self-denial. If a reviewer is up to the job of producing a well-organized and methodical review, which flows well and provides a service to the readership, then it should be possible to be objective in reviewing one's own relevant findings. In reviews written by multiple authors, this may be achieved by assigning the review of the results of a coauthor to different coauthors.

## Rule 10: Be Up-to-Date, but Do Not Forget Older Studies

Given the progressive acceleration in the publication of scientific papers, today's reviews of the literature need awareness not just of the overall direction and achievements of a field of inquiry, but also of the latest studies, so as not to become out-of-date before they have been published. Ideally, a literature review should not identify as a major research gap an issue that has just been addressed in a series of papers in press (the same applies, of course, to older, overlooked studies (“sleeping beauties” [26])). This implies that literature reviewers would do well to keep an eye on electronic lists of papers in press, given that it can take months before these appear in scientific databases. Some reviews declare that they have scanned the literature up to a certain point in time, but given that peer review can be a rather lengthy process, a full search for newly appeared literature at the revision stage may be worthwhile. Assessing the contribution of papers that have just appeared is particularly challenging, because there is little perspective with which to gauge their significance and impact on further research and society.

Inevitably, new papers on the reviewed topic (including independently written literature reviews) will appear from all quarters after the review has been published, so that there may soon be the need for an updated review. But this is the nature of science [27]–[32]. I wish everybody good luck with writing a review of the literature.
